# First Recurrence of Synovial Sarcoma Presenting With Solitary Pancreatic Mass

**DOI:** 10.7759/cureus.26356

**Published:** 2022-06-26

**Authors:** Raja R Narayan, Greg W Charville, Daniel Delitto, Kristen N Ganjoo

**Affiliations:** 1 Surgery, Stanford University School of Medicine, Stanford, USA; 2 Pathology, Stanford University School of Medicine, Stanford, USA; 3 Medical Oncology, Stanford University School of Medicine, Stanford, USA

**Keywords:** bone and soft tissue sarcoma, pancreatic metastasis, chylous leakage, lymph node metastasis, whipple’s pancreaticoduodenectomy

## Abstract

Synovial sarcoma usually presents in the lower extremities and metastasizes to the lungs; however, unusual patterns of recurrence can occur. For patients with recurrent synovial sarcoma to a proximal peripancreatic lymph node, a pancreaticoduodenectomy or Whipple procedure is the best option for a cure. Lymph node metastasis from synovial sarcoma is exceptionally rare, and data guiding the use of the Whipple procedure for curative resection of metastatic synovial sarcoma are even more sparse. In this report, we describe the management of a patient with metastatic synovial sarcoma to a proximal peripancreatic lymph node with a pancreaticoduodenectomy.

## Introduction

Synovial sarcoma is an aggressive mesenchymal neoplasm that metastasizes in 50% of patients [1]. Although the most common site for metastasis is the lung, [1] the disease may occasionally spread to other distant sites including the liver, bone, and, less frequently, the head of the pancreas. For such a rare disease to manifest an even rarer pattern of metastasis, little is known about the biological drivers or anatomic conditions that permit for disease to spread in such an unusual fashion. In this case report, we describe the workup and management for a young woman with a lower extremity synovial sarcoma that developed an unusual peripancreatic lymph node metastasis managed with a pancreaticoduodenectomy.

## Case presentation

An otherwise healthy 42-year-old woman was first found to have a 3.7-cm synovial sarcoma on workup of a lump she found with easy local bruising on the medial aspect of her left upper thigh. A core needle biopsy demonstrated a markedly cellular neoplasm consisting of round and spindle-shaped cells expressing BCL2, patchy CD99, and focal cytokeratin by immunohistochemistry; evidence of SS18 gene rearrangement by break-apart fluorescence in situ hybridization confirmed the diagnosis of monophasic synovial sarcoma. Once radiographic imaging showed no evidence for distant metastasis, the patient underwent a wide local excision yielding a margin-negative 4.5-cm tumor with 25 mitoses per 10 high-power microscopic fields, consistent with FNCLCC grade 3, pT1 disease. She was recommended to undergo adjuvant radiation to reduce the risk for local recurrence, but no systemic therapy was indicated since her tumor was less than 5 cm. She received 31 fractions of 57 Gy for adjuvant radiation. She unfortunately developed severe dysesthesia and motor weakness in the left leg following her operation that improved with physical therapy.

**Figure 1:**
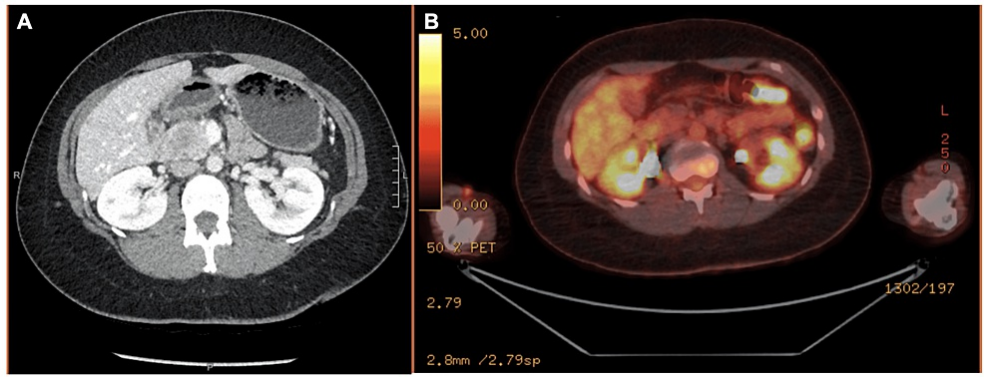
Cross-sectional imaging revealing new pancreatic head mass suspicious for recurrence 1.5 years after wide local resection of left upper thigh mass as noted on (A) CT and (B) FDG-PET. CT, computed tomography; FDG-PET, fluorodeoxyglucose positron emission tomography

After a year and a half, surveillance cross-sectional imaging revealed a new 4.3-cm mass involving the head of the pancreas without any pancreatic ductal dilation or parenchymal atrophy (Figure 1).

**Figure 2:**
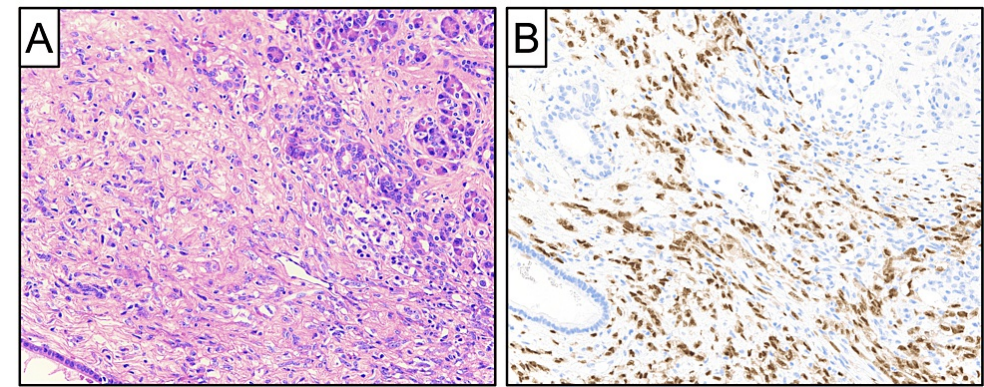
Representative photomicrographs of the pancreaticoduodenectomy, including (A) H&E stain and (B) SS18-SSX fusion-specific immunohistochemistry at 200x magnification, revealing synovial sarcoma involving pancreatic parenchyma. The treated tumor exhibits decreased cellularity and increased stromal hyalinization relative to the pre-treatment tumor.

The patient had also developed new back pain and was referred for endoscopic biopsy, which revealed recurrent synovial sarcoma. She completed four cycles of neoadjuvant adriamycin and ifosfamide ending short of the intended six cycles due to intolerable nausea with vomiting as well as anemia requiring transfusion. On her last preoperative cross-sectional scan, her pancreatic mass had responded down to a size of 2.5 cm. The patient was taken for pancreaticoduodenectomy. Intraoperatively, a large volume of chylous fluid was encountered during the dissection that was controlled with clips and suture ligatures. The patient progressed well postoperatively and was discharged home with her surgical drain on postoperative day 6. Histologic examination of the pancreaticoduodenectomy specimen was notable for a neoplastic proliferation of predominantly spindle-shaped cells, involving pancreatic parenchyma and peripancreatic soft tissue (Figure 2).

The diagnosis of metastatic synovial sarcoma was confirmed by immunohistochemistry using an SS18-SSX fusion-specific antibody [2]. There was histologic evidence of treatment effect, manifesting as hypocellularity and increased stromal hyalinization, relative to the tumor prior to neoadjuvant therapy. One of the 21 lymph nodes evaluated had evidence of metastatic disease, and the focus of sarcoma in the pancreatic head measured 2.6 cm. She continued to progress well as an outpatient, and her drain was removed in the clinic two weeks after her operation.

## Discussion

This case represents an unusual circumstance where a patient with a relatively confined, small synovial sarcoma developed a metastasis to a peripancreatic lymph node. Of the soft tissue sarcomas, synovial sarcoma is an intermediate- to high-grade malignancy for which five-year survival is noted in 36-76% [3]. Three histologic subtypes of synovial sarcoma have been described including monophasic (present in 50-60% displaying only spindle cells), biphasic (present in 20-30% containing features of both epithelial and spindle cells), and poorly differentiated (present in 15-30% with a less archetypal appearance) [4]. Although poorly-differentiated histology is associated with early recurrence and metastasis [4], this patient had a recurrence within two years of their primary excision with monophasic histology.

 At the time of resection, there was extensive lymphatic drainage noted in the process of mobilizing the Whipple specimen. Some chylous fluid can be encountered as part of the Whipple pancreaticoduodenectomy, but a substantially greater volume of lymph was appreciated during the dissection for this patient, which is typically indicative of lymphatic obstruction. This observation in tandem with the odd pattern of metastasis in this patient may bear some relevance; however, this has been poorly defined in prior reports.

Pancreatectomy for metastatic disease is a rare but previously described indication warranted when the pancreatic duct is suspected to be involved by disease. Only seven cases of metastatic synovial sarcoma to the pancreas have been previously reported (Table 1). For peripancreatic lesions minimally abutting the parenchyma, enucleation may be a feasible option in well-selected patients to spare the need for a more extensive pancreaticoduodenectomy. Prior work has described several scenarios through which pancreatectomy can yield long-term survival for patients with malignant disease. In one report analyzing indications for pancreatectomy at a single institution over seven years, the circumstances associated with long-term survival (more than three years) included isolated pancreatic metastasis, absence of prior recurrence, greater than three-year interval between resection of the primary tumor and development of pancreatic metastasis, and if the primary was renal cell carcinoma [5]. The patient in this case met two of these four criteria and was deemed a suitable candidate for pancreatectomy especially given her young age at the time of pancreatic metastasis detection. Future work should seek a larger cohort undergoing resection in a more modern era to best define which patients should be offered pancreatectomy.

**Table 1:** Clinicopathologic characteristics of reported cases with pancreatic metastases from primary synovial sarcoma. DFI, disease-free interval from primary synovial sarcoma diagnosis to date of pancreatic metastasis detection; F female; y years; mo, months; NA, not available; PPPD, pylorus-preserving pancreaticoduodenectomy; PD, pancreaticoduodenectomy; DFS, disease-free survival; OS, overall survival; DP, distal pancreatectomy; DPS, distal pancreatectomy with splenectomy

Author/Year	Age/sex	DFI	Size, cm	Site	More distant metastases	Treatment	Outcome
Yamamoto et al., 2001 [6]	40/F	14 y	NA	Head	No	PPPD	DFS > 6 y
Patel et al., 2006 [7]	44/F	10 y	8	Head	Yes	Bile drainage	NA
Krishna et al., 2014 [8]	37/M	1 y	1.9	Tail	Yes	NA	NA
Makino et al., 2016 [9]	36/M	4 y	3.5	Body	No	Laparoscopic DP	DFS = 30 mo
Lee et al., 2020 [10]	62/F	40 mo	2.7	Body	No	DP	OS = 92 mo
Yokose et al., 2020 [11]	27/F	4 y	3.5	Tail	Yes	Laparoscopic DPS	DFS = 6 mo
Malek et al., 2020 [12]	30/M	15 mo	1.5	Tail	No	DPS	OS = 6 mo
Our case	42/F	30 mo	4.3	Head	No	PD	DFS = 1 mo

## Conclusions

Pancreatic metastasis from primary synovial sarcoma is exceedingly rare but manageable by pancreatectomy. If the tumor biology is not indicative of aggressive pathology, the rare event of lymph node metastasis may occur in patients with atypical lymphatic anatomy and may also be associated with higher volume chylous drainage noted during the surgical dissection. Further work is needed to better characterize the pathophysiology for uncommon patterns of metastasis as well as the indications of pancreatic metastasectomy.
